# Study of the Knowledge, Attitudes and Practices of Students from Medical Majors Regarding HPV Infections and HPV Vaccines

**DOI:** 10.3390/pathogens14121270

**Published:** 2025-12-11

**Authors:** Meri Hristamyan, Vanya Rangelova, Theodor Lolovski, Meysam Homadi, Ani Kevorkyan

**Affiliations:** 1 Department of Epidemiology and Disaster Medicine, Faculty of Public Health, Medical University of Plovdiv, 4002 Plovdiv, Bulgaria; 2 Medical Faculty, Medical University of Plovdiv, 4002 Plovdiv, Bulgaria

**Keywords:** HPV, HPV infection, HPV vaccine, knowledge, attitudes, vaccination, practices

## Abstract

Background: Human papillomavirus (HPV) is a highly prevalent sexually transmitted infection that can lead to cervical and other anogenital and oropharyngeal cancers. Despite available vaccines, vaccination coverage remains low in Bulgaria. This study aimes to assess the knowledge, attitudes, and practices of Medical University students in HPV prevention. Materials: A cross-sectional anonymous survey was conducted at the Medical University-Plovdiv, Bulgaria. Results: A total of 1485 students, primarily women (60.1%) with a median age of 22.78 years, participated. Four hundred fifty-two (30.4%) reported having received the HPV vaccine. Of the unvaccinated, 800 (77.8%) expressed willingness to receive the vaccine. Vaccinated respondents were more likely to report having had five or more sexual partners (37.1%) compared to unvaccinated respondents (21.1%) (χ^2^ = 77.136, *p* < 0.001). Approximately one-third (36.4%) mistakenly believe condoms provide complete protection and that antibiotics effectively treat HPV. Students who opposed the assertion that vaccinating minors suggests early sexual activity is permissible were 1.89 times more likely to be vaccinated. Conclusions: Medical University students possess insufficient understanding of HPV transmission, health outcomes, and prevention. Their attitudes and practices require improvement. Enhancing the curriculum with comprehensive HPV information will better equip future healthcare providers and improve public health outcomes.

## 1. Introduction

Human papillomavirus (HPV) is a highly prevalent sexually transmitted infection (STI) with significant implications for public health, since 85–90% of sexually active people will acquire it at some point throughout their life [1,2]. HPV is a DNA virus with over 200 identified types, categorised into low-risk (lr-HPV) and high-risk (hr-HPV) HPV types based on their potential to cause disease [1], and is primarily transmitted through sexual contact, although some non-sexual routes have also been suggested [3,4].

Among the 14 classified hr-HPVs (HPV 16, 18, 31, 33, 35, 39, 45, 51, 52, 56, 58, 59, 66, and 68), types 16 and 18 are the most oncogenic, responsible for approximately 70% of cervical cancers, while other hr-HPVs, such as types 31, 33, 45, 52, and 58, contribute to an additional 15–20% of cases [4,5]. Low-risk HPV types, including HPV-6 and HPV-11, do not cause cancer but are linked to benign conditions such as genital warts and recurrent respiratory papillomatosis (RRP), which affects the respiratory tract [4]. Vaginal, vulvar, penile, and some head and neck cancers are associated with hr-HPV types [6]. Furthermore, oropharyngeal cancer, increasingly prevalent in men, is strongly associated with HPV-16 [7], and approximately 90% of anal cancers are also linked to hr-HPVs [8]. High-risk HPV types mediate oncogenesis primarily through E6 and E7 oncoproteins, which inactivate tumour suppressors p53 and Rb, respectively, promoting genomic instability, cell cycle deregulation, and malignant transformation across anogenital and oropharyngeal sites. These oncoproteins disrupt normal cellular checkpoints, enabling persistent viral replication in differentiated keratinocytes and accumulation of secondary genetic alterations essential for cancer progression [8].

In 2022, HPV-associated cancers accounted for 1,505,394 new cases worldwide (922,728 in females and 582,666 in males), with an age-standardised incidence rate (ASIR) of 20.9 per 100,000 people, and 755,303 deaths (ASMR of 10.2 per 100,000). Cervical cancer remains the predominant HPV-related malignancy, comprising over 90% of cases in women, though head and neck cancers represent the largest absolute number of cases (685,204) [9]. These figures underscore the persistent global challenge despite vaccination efforts. In asymptomatic women, the prevalence of cervical HPV infection globally is approximately 11%, with rural women and those attending cervical cancer screening clinics having higher prevalence rates compared to urban populations [10]. The global pooled prevalence of genital HPV infection among men is 31% for any HPV type and 21% for hr-HPVs, and prevalence peaks are observed in young adults aged 25–29 years [11]. Although the majority of HPV-related malignancies are cervical, there is an escalating epidemic of HPV-driven oropharyngeal squamous cell carcinoma in specific regions globally, especially low- and middle-income countries (LMICs) [1].

Bulgaria, a country located in Eastern Europe, has a population of 6.4 million, of whom 3.06 million are women aged 15 years and older who are at risk of developing cervical cancer. Estimates from 2023 show that 1009 women receive a cervical cancer diagnosis each year, and 503 of them succumb to the disease. Approximately 9.7% of women in the general population are expected to be infected with cervical HPV-16/18 at any given time, and 84.7% of invasive cervical malignancies are linked to HPVs 16 or 18 [12].

On a global scale, six HPV vaccines are officially licenced for use: three bivalent, two quadrivalent, and one nonavalent. The most frequently employed are bivalent, which targets HPV-16 and 18, with some cross-protection against types 31 and 45; quadrivalent, which provides protection against HPV types 6, 11, 16, and 18; and nonavalent, which expands coverage to five additional types—31, 33, 45, 52, and 58—offering nearly complete protection against HPV-related infections and precancers [13,14].

Widespread vaccination could potentially eliminate cervical cancer, as demonstrated by countries with high vaccination rates like the UK and Australia [15,16,17]. The WHO emphasises the importance of HPV vaccination for both genders, focusing especially on regions with low female coverage, aiming to vaccinate 90% of girls by age 15 and promoting the inclusion of boys in immunisation efforts [18]. Several models have indicated that vaccinating both males and females is more effective at reducing HPV infections and related diseases than focusing vaccination efforts solely on girls [19].

Despite the effectiveness of vaccines, many regions face challenges linked to misconceptions about vaccination and low general HPV knowledge and therefore have low vaccination rates [15]. As of 2023, fewer than 15% of females globally have completed the full course of HPV vaccination, while vaccination coverage among males lags even further behind, with only around 4% of men worldwide having received full HPV immunisation as of 2019 [20,21]. Bulgaria expanded its immunisation coverage by providing the HPV vaccine free of charge for boys aged 10–13 years in response to the very low immunisation coverage of 2% and following international recommendations in April 2025 [22].

Raising HPV vaccination rates is a public health priority that requires a multifaceted approach, including awareness campaigns, educational initiatives, and proper training for healthcare professionals [23]. Enhancing the knowledge, attitudes and practices of university students from medical majors is especially important, as they play a vital role in promoting HPV prevention, raising public awareness, and increasing vaccine acceptance in the future.

## 2. Materials and Methods

### 2.1. Study Design and Settings

A cross-sectional anonymous survey was conducted in the period May–October 2023 among students at the Medical University of Plovdiv, Bulgaria. The survey sought to assess knowledge, attitudes, and practices about HPV infection and vaccination. The survey was disseminated electronically through Google Forms via the university’s email and correspondence system. No personal information was gathered. Before commencing the questionnaire, all participants provided informed consent, affirming their readiness to participate.

### 2.2. Questionnaire

The questionnaire was designed in both Bulgarian and English, considering that university studies are undertaken in these languages. The study had four panels: demographics, knowledge of HPV infections and the HPV vaccine, attitudes towards the HPV vaccine, and practices concerning the HPV vaccine. The enquiries assessing student attitudes utilised a 5-point Likert scale, ranging from 0 (strongly disagree) to 5 (strongly agree).

### 2.3. Statistical Analysis

Descriptive analyses were applied to both quantitative and qualitative data. For continuous variables, results were expressed as means with standard deviations or, in cases of non-normal distribution, as medians accompanied by interquartile ranges (25th and 75th percentiles). Categorical variables were summarised using counts and percentages (n, %). The Kolmogorov–Smirnov test was used to examine whether continuous data followed a normal distribution.

Group comparisons for non-normally distributed continuous variables were performed using the Mann–Whitney U test, while differences in proportions between two groups were evaluated using the z-test. Variables demonstrating a *p*-value below 0.10 in univariate analyses were included in a binary logistic regression model to assess the likelihood of a given observation falling into one of two outcome categories—specifically, HPV vaccination—based on several predictor variables. The model yielded odds ratios (ORs) with corresponding 95% confidence intervals (CIs) for each predictor.

All data management, statistical processing, and analysis were carried out using IBM SPSS Statistics for Windows, version 25.0 (IBM Corp., Armonk, NY, USA; released 2017).

## 3. Results

A total of 1485 students, predominantly women (60.1%) with a median age of 22.78 years, took part in the survey. Bulgarian nationals represented 58.6% of respondents (n = 870), while 41.4% (n = 615) were international students. A detailed breakdown of socio-demographic characteristics is presented in Table 1.

Of all surveyed students, 452 (30.4%) reported having received the HPV vaccine. Among those unvaccinated, 800 (77.8%) expressed willingness to receive the vaccine if provided the opportunity.

A statistically significant difference in vaccination rates was noted by nationality: 50.6% of international students (n = 311) were vaccinated, in contrast to 16.2% of Bulgarian students (n = 141) (χ^2^ = 201.787, *p* < 0.001). Significant gender disparities were observed, with a greater percentage of women (n = 319, 70.6%) vaccinated in contrast to men (n = 129, 22.3%) (χ^2^ = 29.978, *p* < 0.001).

Individuals vaccinated against HPV were more likely to report having had five or more sexual partners (n = 127, 37.1%) compared to unvaccinated individuals (n = 215, 21.1%) (χ^2^ = 77.136, *p* < 0.001). Furthermore, approximately 43.8% of vaccinated students (n = 67) had both parents employed in healthcare, in contrast to 26.1% (n = 86) of the unvaccinated cohort (χ^2^ = 26.944, *p* < 0.001).

Vaccination rates correlated positively with the year of study: 42.3% (n = 107) of students in their third year or higher were vaccinated, while students in previous years were largely unvaccinated (χ^2^ = 69.443, *p* < 0.001). Age (*p* = 0.062) and sexual preferences (*p* = 0.110) were not substantially correlated with HPV vaccination status, indicating these factors had minimal impact within this group.

The participants demonstrate adequate understanding of HPV infections in some commonly recognised aspects, yet they exhibit gaps in other areas. Table 2 provides a comprehensive breakdown of knowledge regarding HPV infection.

Nearly all participants, regardless of their vaccination status, acknowledge that HPV is transmitted through sexual contact and that infection with the virus increases the risk of cervical cancer. The awareness of vulvar and penile cancer risk stands at 84.9% (n = 1261), showing a slight decline. This trend continues with anal cancer awareness at 71.2% (n = 1058) and oropharyngeal cancer awareness at 74.5% (n = 1106).

A minority of students from medical majors, specifically 46.9% (n = 697), hold the belief in complete recovery from HPV infection. Additionally, approximately one-third of these students, 36.4% (n = 541), mistakenly think that condoms provide complete protection against HPV and that antibiotics can effectively treat viral infections.

It is noteworthy that a higher number of the vaccinated participants inaccurately claim that HPV infection typically presents with visible symptoms (n = 165, 36.5%) (χ^2^ = 9.287, *p* = 0.02).

When assessing the knowledge of the respondents regarding the HPV vaccine, we discovered some intriguing results (Table 3). A notably greater percentage of vaccinated participants indicate that a family member has been diagnosed with cervical cancer or other HPV-related oncological diseases (n = 77, 17.0%) in contrast to non-vaccinated respondents (n = 89, 8.6%) (χ^2^ = 22.450, *p* < 0.001).

Approximately 47.1% of vaccinated respondents (n = 213) are aware that the vaccine can be administered to individuals with an existing HPV infection, in contrast to 35.7% of non-vaccinated respondents (n = 369), indicating a statistically significant association (χ^2^ = 17.154, *p* < 0.001). This finding is supported by a higher proportion of non-vaccinated students who believe that screening is necessary prior to vaccination (72.9%, n = 753 non-vaccinated vs. 55.3%, n = 250 vaccinated students) (χ^2^ = 44.350, *p* < 0.001).

We further studied the attitudes of the students regarding HPV vaccines (Table 4). A significant majority of students agree that vaccines are important in general (n = 1280, 86.2%) and that HPV vaccines are particularly necessary/beneficial (n = 1194, 80.4%). The level of agreement on this matter is notably greater among vaccinated respondents (χ^2^ = 201.787, *p* < 0.001). Conversely, unvaccinated participants (n = 667, 64.6%) more frequently agree with the claim that a rise in HPV-related diseases might occur in the near future if vaccination rates remain low compared with vaccinated students (n = 262, 58%) (χ^2^ = 10.806, *p* = 0.029).

Approximately 21.5% (n = 319) indicate that vaccine price may influence their decision, and a comparable proportion would only receive vaccination if it were provided at no cost. A significantly higher majority of vaccinated respondents (n = 397, 87.8%) would recommend vaccination to family members, friends, or future patients, compared to non-vaccinated students (n = 696, 69.3%) (χ^2^ = 117.928, *p* < 0.001). A majority of respondents (n = 1200, 80.8%) acknowledge the necessity for enhanced awareness regarding HPV infections and vaccines, with a desire for increased knowledge (n = 1142, 76.9%), indicating significant recognition of educational deficiencies.

We conducted a logistic regression analysis to assess the impact on the probability of receiving the HPV vaccine (Table 5). The logistic regression model demonstrated statistical significance, χ^2^(3) = 708.414, *p* < 0.001. The model correctly identified 53.8% of the cases. Regarding demographic parameters, respondents in their sixth year of study were 0.385 times less likely to have received the HPV vaccine, while female students were 5.1 times more likely to have been vaccinated. Respondents who expressed fear of potential adverse outcomes following HPV vaccination were less likely to have received the vaccine. Conversely, students who disagreed with the claim that offering the HPV vaccine to minors implies that early sexual activity is acceptable were 1.89 times more likely to have had the vaccination.

## 4. Discussion

Concerning understanding of HPV infection, we discovered that sexual transmission and cervical cancer are the most often recognised facts concerning HPV. Further research among health sciences students also confirms a significant awareness of these factors [24,25,26,27]. A diminished awareness of the relationship between HPV and non-cervical malignancies, particularly oropharyngeal and anal cancers, leads to an underappreciation of the virus’s extensive carcinogenic potential. In the United States, merely one-third of participants acknowledged the association between HPV and oropharyngeal cancer, while in Saudi Arabia, dental students exhibited an average knowledge score of 0.9 ± 1.2 out of 5 regarding oropharyngeal cancer, indicating a significantly lower level of awareness compared to our study [28,29]. Furthermore, hardly 50% of medical students in a separate study were aware of the range of HPV-associated malignancies [30]. In a similar study, nursing students in Italy exhibited a limited understanding of different cancers associated with the virus [31].

Concerning the transmission of HPVs, researchers report similar findings, identifying vaginal contact as the predominant known route, while observing decreased awareness of anal, oral, and vertical transmission [23,32]. A cross-sectional survey of medical and paramedical students in India revealed that only approximately 35% accurately recognised vertical transmission (from mother to foetus) as a potential route of HPV infection, whereas many erroneously believed in transmission through blood or contaminated food [33]. Medical students typically have a better overall understanding of HPV transmission pathways compared to dental and nursing students, presumably due to enhanced clinical exposure. Although vaginal intercourse is widely acknowledged as a transmission route, some students underestimate the risks associated with oral and anal sex, which are significant for HPV-related head and neck cancers as well as anal cancers. This disparity may influence their future clinical counselling and vaccination promotion [34,35].

Only fifty percent of the surveyed students believe in total recovery from HPV infection; one-third erroneously assume that condoms provide complete protection, and others believe that antibiotics can treat HPV [36]. Additional studies indicate misunderstandings regarding HPV infection and prevention, such as a survey conducted among medical students in China, where only one-third are aware that HPV infection can be cured or resolved spontaneously. Approximately one-third of college students in a separate Chinese survey falsely believe that antibiotics may eradicate HPV, indicating a notable misperception [37]. This misperception presumably arises from a misunderstanding of viral and bacterial illnesses. This misperception presumably arises from a misunderstanding of viral and bacterial illnesses. A Hungarian nationwide poll targeting college students and their parents revealed that 38% believe condoms offer complete protection against HPV, which aligns with our findings [38].

Approximately one-third of participants were vaccinated, and among those who were unvaccinated, the majority expressed a willingness to receive the vaccine if possible. Daniel et al. [39] similarly report that 32.1% of medical students have completed the full HPV vaccine series, while in Brazil, the vaccination rate is lower at 21.1%, with most students already being sexually active [40]. Mulenga et al. reported that only 6.5% of pharmacy students had received the vaccine [41]. Comparable vaccination rates were noted among medical and paramedical Indian students (6.8%) [42], while only 3.6% of female university students were in health schools in Jordan [43].

The present study reveals a higher vaccination rate among females, consistent with the existing literature indicating that females are more likely to receive HPV vaccinations than males. This trend is likely attributable to the established link between HPV and cervical cancer, along with regional vaccination guidelines that consider gender. Numerous studies indicate notable gender disparities in knowledge and uptake of HPV vaccination [44,45].

We observed that vaccinated participants are significantly more likely to have a family member diagnosed with cervical cancer or HPV-related cancer and have a family member who is also vaccinated, suggesting a particularly strong familial influence in our sample. Furthermore, the interconnectedness of parents’ willingness to vaccinate themselves and their children suggests that vaccination often clusters within families [40,46].

Over 70% of our overall participants, along with the majority of vaccinated medical students in India, exhibit comparable findings, with 68.3% expressing a willingness to recommend the vaccine to others [24]. Vaccinated medical students typically achieve higher scores on knowledge assessments and report greater comfort with patient counselling [47]. Furthermore, dental students consider the endorsement of HPV vaccination to be part of their professional obligations [35].

Our study did not find embarrassment to be a significant barrier to HPV vaccination; however, existing research highlights that feelings of stigma or shame may hinder individuals from disclosing their vaccination status and receiving the vaccine, indicating that this factor can adversely affect vaccine uptake [48,49,50]. A 2023 study examining university students identified embarrassment associated with HPV vaccination as a notable psychosocial barrier. This issue was particularly linked to the vaccine’s connection to sexual activity, with a greater impact observed among female students and those possessing limited knowledge about HPV [51].

The majority of participants in our study acknowledged the necessity for enhanced awareness and information about HPV and the available vaccines, indicating a desire to broaden their knowledge of the subject. A cross-sectional survey conducted in India revealed that a considerable percentage of health sciences students expressed a desire for increased education and training regarding HPV infection and vaccination [24]. Similarly, in Jordan, more than 70% of participants indicated a need for additional information and formal education about HPV and its vaccine to feel confident in recommending it [43]. A study in China highlights the considerable influence of HPV knowledge on students’ vaccination decisions [52]. Evidence from an educational intervention aimed at female healthcare students indicates that structured health education programmes enhance knowledge and awareness of HPV, subsequently increasing the likelihood of vaccination [53].

The findings from the study underscore the importance of implementing educational interventions to improve HPV knowledge and reduce vaccine hesitancy among undergraduate students in medical universities. HPV content should be integrated into preclinical subjects such as microbiology, virology, and infectious diseases curricula to build foundational knowledge of transmission, oncogenesis, and prevention among medical students. Additionally, clinical rotations in gynaecology, oncology, and primary care should incorporate team-based modules with case vignettes on vaccination counselling to improve attitudes and practices. It would be beneficial to explore the application of technological advancements, including mobile applications and virtual reality, to improve HPV education and engagement among students and the broader population [54,55,56,57,58].

## 5. Conclusions

This study reveals that university students in medical specialities possess insufficient knowledge of HPV in various clinical domains, specifically its transmission routes, related health complications, and preventive strategies, including vaccination. Their attitudes and practices regarding HPV immunisation require improvement. Given that healthcare professionals are essential for patient education and public health advocacy, it is imperative to rectify these deficiencies through comprehensive and systematically organised medical education, thereby enhancing their understanding of the issue and fostering improved attitudes and practices regarding HPV vaccines. Enhancing the curriculum with comprehensive information on HPV, its epidemiology, and preventive measures will better equip future healthcare providers to promote awareness and preventive initiatives for improved public health outcomes.

## Figures and Tables

**Table 1:** Socio-demographic characteristics of the study respondents.

Variable	All Respondents (n = 1485)	HPV-Vaccinated (n = 452)	HPV-Non-Vaccinated (n = 1033)	*p*-Value
**Age**, **mean ± Std**	22.78 ± 3.143	22.71 ± 2.292	22.82 ± 2.292	0.062
**Gender, n (%)**				**<0.001**
Male	579 (39.0)	129 (22.3)	450 (77.7)	
Female	893 (60.1)	319 (35.7)	574 (64.3)	
Other	13 (0.9)	4 (30.8)	9 (69.2)	
**Sexual preferences**				0.110
Heterosexual	1377 (92.7)	424 (30.8)	953 (69.2)	
Homosexual	53 (3.6)	9 (17.0)	44 (83.0)	
Bisexual	39 (2.6)	15 (38.5)	24 (61.5)	
Other	16 (1.1)	4 (25.0)	12 (75.0)	
**Marital status**				**0.011**
Long-term partner	607 (40.9)	159 (26.2)	444 (73.8)	
In a non-committed relationship	207 (13.9)	66 (31.9)	141 (68.1)	
No partner	671 (45.2)	227 (33.8)	448 (66.2)	
**Number of sexual partners for the last 12 months**				**<0.001**
5 or more	342 (23.0)	127 (37.1)	215 (62.9)	
2–4	673 (45.3)	151 (22.4)	522 (77.6)	
1	165 (11.1)	31 (18.8)	134 (81.2)	
none	305 (20.6)	143 (46.9)	162 (53.1)	
**Parent who is a healthcare worker**				**<0.001**
Yes, both	153 (10.3)	67 (43.8)	86 (56.2)	
Yes, one of them	368 (24.8)	133 (36.1)	235 (63.9)	
No	964 (64.9)	252 (26.1)	712 (73.9)	
**Main language of studies, n (%)**				**<0.001**
Bulgarian	870 (58.6)	141 (16.2)	729 (83.8)	
English	615 (41.4)	311 (50.6)	304 (49.4)	
**Main subject of studies, n (%)**				**<0.001**
Medicine	872 (58.7)	298 (34.1)	574 (65.9)	
Dental Medicine	286 (19.2)	87 (30.4)	199 (69.6)	
Pharmacy	155 (10.4)	39 (25.2)	116 (74.8)	
Medical college	172 (11.7)	28 (16.2)	144 (83.8)	
**Year of study, n (%)**				**<0.001**
1st year	69 (4.6)	10 (14.5)	59 (85.5)	
2nd year	203 (13.7)	32 (15.8)	171 (84.2)	
3rd year	253 (17.0)	107 (42.3)	146 (57.7)	
4th year	246 (16.6)	81 (33.0)	165 (67.0)	
5th year	418 (28.1)	154 (36.8)	264 (63.2)	
6th year	296 (20.0)	68 (23.0)	228 (77.0)	

**Table 2:** Knowledge regarding HPV infection.

Question	All Respondents (n = 1485)	HPV-Vaccinated (n = 452)	HPV-Non-Vaccinated (n = 1033)	*p*-Value
	**Respondents that responded affirmatively (yes)**			
**HPV is transmitted by sexual contact**	1454 (97.9)	443 (98.0)	1011 (97.9)	0.864
**HPV infection is common**	1213 (81.7)	382 (84.5)	831 (80.4)	0.062
**Males are only carriers of HPV (without clinical manifestation)**	462 (31.1)	141 (31.2)	321 (31.1)	0.963
**HPV infection increases the risk for cervical cancer**	1409 (94.9)	429 (94.9)	980 (94.9)	0.973
**HPV infection increases the risk for anal cancer**	1058 (71.2)	336 (74.3)	722 (69.9)	0.082
**HPV infection increases the risk for oropharyngeal, laryngeal and oral cancer**	1106 (74.5)	348 (77.0)	758 (73.4)	0.142
**HPV infection increases the risk for vulvar and penile cancer**	1261 (84.9)	374 (82.7)	887 (85.9)	0.122
**HPV infection increases the risk for genital warts**	1328 (89.4)	411 (90.9)	917 (88.8)	0.213
**The PAP test can identify HPV infection**	1199 (80.7)	377 (83.4)	822 (79.6)	0.085
**The number of sexual partners affects the risk of HPV infection**	1377 (92.7)	417 (92.3)	960 (93.0)	0.644
**Condoms can completely protect a person from HPV infection**	541 (36.4)	166 (36.7)	375 (36.3)	0.876
**A person can completely recover from HPV infection**	697 (46.9)	227 (50.2)	470 (45.5)	0.093
**HPV infection is treated successfully with antibiotics**	474 (31.9)	147 (32.5)	327 (31.7)	0.742
**Most people with HPV infection have visible symptoms**	460 (31.0)	165 (36.5)	295 (28.5)	**0.02**
**You can get infected with HPV during vaginal sexual contact**	1442 (97.1)	435 (96.2)	1007 (97.5)	0.188
**You can get infected with HPV during anal sexual contact**	1247 (84.0)	386 (85.4)	861 (83.3)	0.322
**You can get infected with HPV during oral sexual contact**	1215 (81.8)	378 (83.6)	837 (81.0)	0.232
**There is a possibility for vertical transmission of HPV from mother to child**	1111 (74.8)	341 (75.4)	770 (74.5)	0.712
**The early start of sexual life is a risk factor for HPV infection**	1063 (71.6)	338 (74.8)	725 (70.2)	0.071

**Table 3:** Knowledge regarding HPV vaccines.

Question	All Respondents (n = 1485)	HPV-Vaccinated (n = 452)	HPV-Non-Vaccinated (n = 1033)	*p*-Value
	**Respondents that responded affirmatively (yes)**			
**There is a person in my family diagnosed with cervical cancer or other HPV-associated oncological disease**	166 (11.2)	77 (17.0)	89 (8.6)	**<0.001**
**I knew about HPV vaccines before this study**	1277 (86.0)	429 (95.0)	848 (82.1)	**<0.001**
**There is a person in my family vaccinated with the HPV vaccine**	490 (33.0)	344 (76.1)	146 (14.1)	**<0.001**
**HPV vaccination is recommended only for pre-pubertal children**	333 (22.4)	122 (27.0)	211 (20.4)	**0.005**
**The HPV vaccine can be administered to already sexually active people**	1162 (78.2)	371 (82.1)	791 (76.6)	**0.018**
**The HPV vaccine can be administered to a person with a present HPV infection**	582 (39.2)	213 (47.1)	369 (35.7)	**<0.001**
**You should have an HPV screening before receiving the vaccine**	1003 (67.5)	250 (55.3)	753 (72.9)	**<0.001**
**It’s safe to have multiple sexual partners after a full course of HPV vaccination**	335 (22.6)	124 (27.4)	211 (20.4)	**0.003**
**Women already vaccinated with an HPV vaccine don’t need HPV screening for cervical cancer**	215 (14.5)	79 (17.5)	136 (9.2)	**0.030**

**Table 4:** Attitudes towards HPV vaccines.

Question	All Respondents (n = 1485)	HPV-Vaccinated (n = 452)	HPV-Non-Vaccinated (n = 1033)	*p*-Value
	**Respondents that responded affirmatively (agree/strongly agree)**			
**Vaccines are necessary in general**	1280 (86.2)	407 (90.0)	873 (84.2)	**0.04**
**HPV vaccines are highly necessary/beneficial**	1194 (80.4)	409 (90.5)	785 (76.0)	**<0.001**
**Only women need the HPV vaccine**	182 (12.2)	64 (14.1)	118 (11.4)	0.139
**The HPV vaccine is only for people with multiple sex partners**	154 (10.4)	45 (9.9)	109 (10.6)	0.729
**There will be an increased trend for HPV-related diseases in the near future if vaccination rates are low**	929 (62.6)	262 (58.0)	667 (64.6)	**0.029**
**The HPV vaccine may affect reproductive health**	535 (36.0)	176 (38.9)	359 (34.7)	0.122
**The HPV vaccine may cause neurological and other adverse reactions/complications**	388 (26.1)	128 (28.3)	260 (25.2)	0.204
**I am not at risk for HPV infection**	517 (34.8)	180 (39.8)	337 (32.6)	0.07
**I would be ashamed to ask to be vaccinated against**	144 (9.7)	44 (9.7)	100 (9.7)	0.974
**The price of HPV vaccines would influence my decision to get vaccinated**	319 (21.5)	95 (21.0)	224 (21.7)	0.773
**I would only get vaccinated against HPV if it was free.**	319 (21.5)	108 (23.9)	211 (20.4)	0.134
**There is a need for better awareness about HPV and HPV-related diseases and vaccines**	1200 (80.8)	358 (79.2)	842 (81.5)	0.299
**I would advise family members/friends/future patients to get vaccinated against HPV**	1093 (73.6)	397 (87.8)	696 (67.3)	**<0.001**
**I want to gain more knowledge about HPV infection and HPV vaccines**	1142 (76.9)	329 (72.8)	813 (78.7)	**0.013**

**Table 5:** Binominal logistic regression model for HPV vaccine uptake.

Model	Unstandardised Coefficients		Wald	df	Sig.	Exp (B)	95% Confidence Interval for B	
	B	Std. Error					Lower Bound	Upper Bound
(Constant)								
**Gender**								
Female gender (baseline)								
Male gender	0.058	0.859	0.004	1	0.947	1.059	0.197	5.706
Other gender	1.481	0.861	2.957	1	0.086	4.396	0.813	23.766
**Main subject of studies**								
Medical college (baseline)								
Medicine	0.520	0.303	2.945	1	0.086	1.683	0.929	3.049
Dental medicine	0.723	0.332	4.747	1	**0.029**	2.061	1.075	3.949
Pharmacy	0.605	0.373	2.626	1	0.105	1.832	0.881	3.808
**Parent who is a healthcare worker**								
Both parents (baseline)								
None	−0.853	0.245	12.135	1	**0.000**	0.426	0.264	0.689
One of the parents	−0.584	0.268	4.743	1	**0.029**	0.558	0.330	0.943
**HPV infection is common**								
Yes (baseline								
No	−0.084	0.208	0.163	1	0.687	0.919	0.611	1.383
**There is a person in my family diagnosed with cervical cancer or other HPV-associated oncological disease**								
Yes (baseline)								
No	−0.383	0.242	2.503	1	0.114	0.682	0.424	1.096
**There is a person in my family vaccinated with the HPV vaccine**								
Yes (baseline)								
No	−3.043	0.170	319.740	1	**0.000**	0.048	0.034	0.067
**HPV vaccination is recommended only for pre-pubertal children**								
Yes (baseline)								
No	−0.353	0.182	3.756	1	0.053	0.702	0.491	1.004
**The HPV vaccine can be administered to already sexually active people**								
Yes (baseline								
No	−0.539	0.160	11.338	1	0.001	0.583	0.426	0.798
**Vaccines are necessary in general**								
Strongly disagree (baseline)								
Strongly agree	−1.909	0.779	6.015	1	**0.014**	0.148	0.32	0.681
Agree	−1.823	0.795	5.263	1	**0.022**	0.161	0.034	0.767
Neither agree nor disagree	−1.526	0.801	3.629	1	0.057	0.217	0.045	1.045
Disagree	−1.527	1.029	2.204	1	0.138	0.217	0.029	1.631
**HPV vaccines are highly necessary/beneficial**								
Strongly disagree (baseline)								
Strongly agree	2.449	1.030	5.652	1	**0.017**	11.578	1.537	87.209
Agree	1.768	1.032	2.932	1	0.087	5.859	0.774	44.326
Neither agree nor disagree	0.507	1.037	0.239	1	0.625	1.661	0.217	12.680
Disagree	1.177	1.180	0.994	1	0.319	3.244	0.321	32.803
**Only women need the HPV vaccine**								
Strongly disagree (baseline)								
Strongly agree	0.466	0.387	1.454	1	0.228	1.594	0.747	3.400
Agree	0.371	0.348	1.137	1	0.286	1.450	0.732	2.869
Neither agree nor disagree	0.411	0.236	3.037	1	0.081	1.508	0.950	2.393
Disagree	0.273	0.211	1.682	1	0.195	1.314	0.870	1.987
**The HPV vaccine may affect reproductive health**								
Strongly disagree (baseline)								
Strongly agree	0.100	0.257	0.151	1	0.697	1.105	0.668	1.830
Agree	−0.196	0.309	0.405	1	0.524	0.822	0.449	1.504
Neither agree nor disagree	0.127	0.263	0.232	1	0.630	1.135	0.678	1.900
Disagree	0.164	0.278	0.347	1	0.556	1.178	0.683	2.031
**There is a need for better awareness about HPV and HPV-related diseases and vaccines**								
Strongly disagree (baseline)								
Strongly agree	0.816	0.593	1.896	1	0.168	2.261	0.708	7.224
Agree	0.504	0.611	0.680	1	0.410	1.655	0.499	5.488
Neither agree nor disagree	0.743	0.634	1.373	1	0.241	2.103	0.606	7.293
Disagree	1.557	0.723	4.638	1	**0.031**	4.742	1.150	19.552
**I want to gain more knowledge about HPV infection and HPV vaccines**								
Strongly disagree (baseline)								
Strongly agree	−1.236	0.670	3.406	1	0.065	0.291	0.078	1.080
Agree	−0.1090	0.675	2.608	1	0.106	0.336	0.090	1.262
Neither agree nor disagree	−0.634	0.684	0.860	1	0.354	0.530	0.139	2.027
Disagree	−0.243	0.798	0.093	1	0.760	0.784	0.164	3.744

## Data Availability

Data will be provided on request.
